# Supplementary material for: Host-parasite tissue adhesion by a secreted type of β-1,4-glucanase in the parasitic plant Phtheirospermum japonicum
Source: Commun Biol. 2020 Jul 30;3:407. doi: 10.1038/s42003-020-01143-5 (PMC7393376; doi:10.1038/s42003-020-01143-5)
Supplement: Supplementary file 1 — Supplementary Information [file 42003_2020_1143_MOESM1_ESM.pdf]

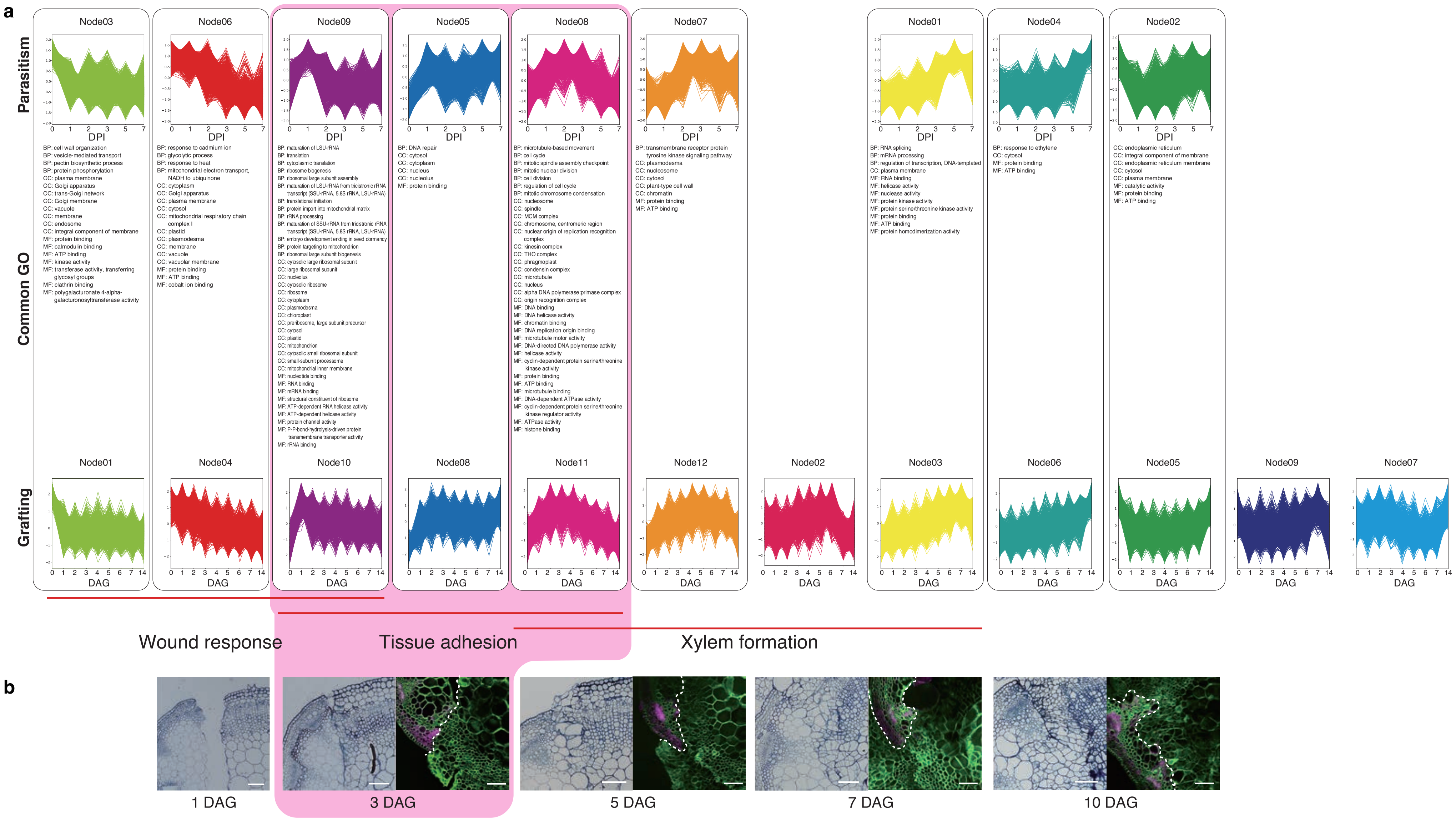

Supplementary Fig. 1: Whole self-organizing map (SOM) clusters and gene ontology (GO) terms for parasitism and grafting between *P. japonicum* and *Arabidopsis*. **a** SOM clusters with similar patterns in parasitism (top) and grafting (bottom) are shown. GO terms commonly found in the parasitism and grafting were listed among clusters. BP, CC and MF stand for GO-subcategory of biological process, cellular components and molecular function, respectively. 20 GOs with a *P* value of 0.05 or less were selected in each category. **b** Tissue sections of the graft junction are shown. Fluorescence images of the graft junction are also shown where *P. japonicum* was grafted to *Arabidopsis RPS5a::LTI6b-tdTomato*. Green indicates the cell wall, magenta indicates tdTomato fluorescence. Scale bars, 100 µm.

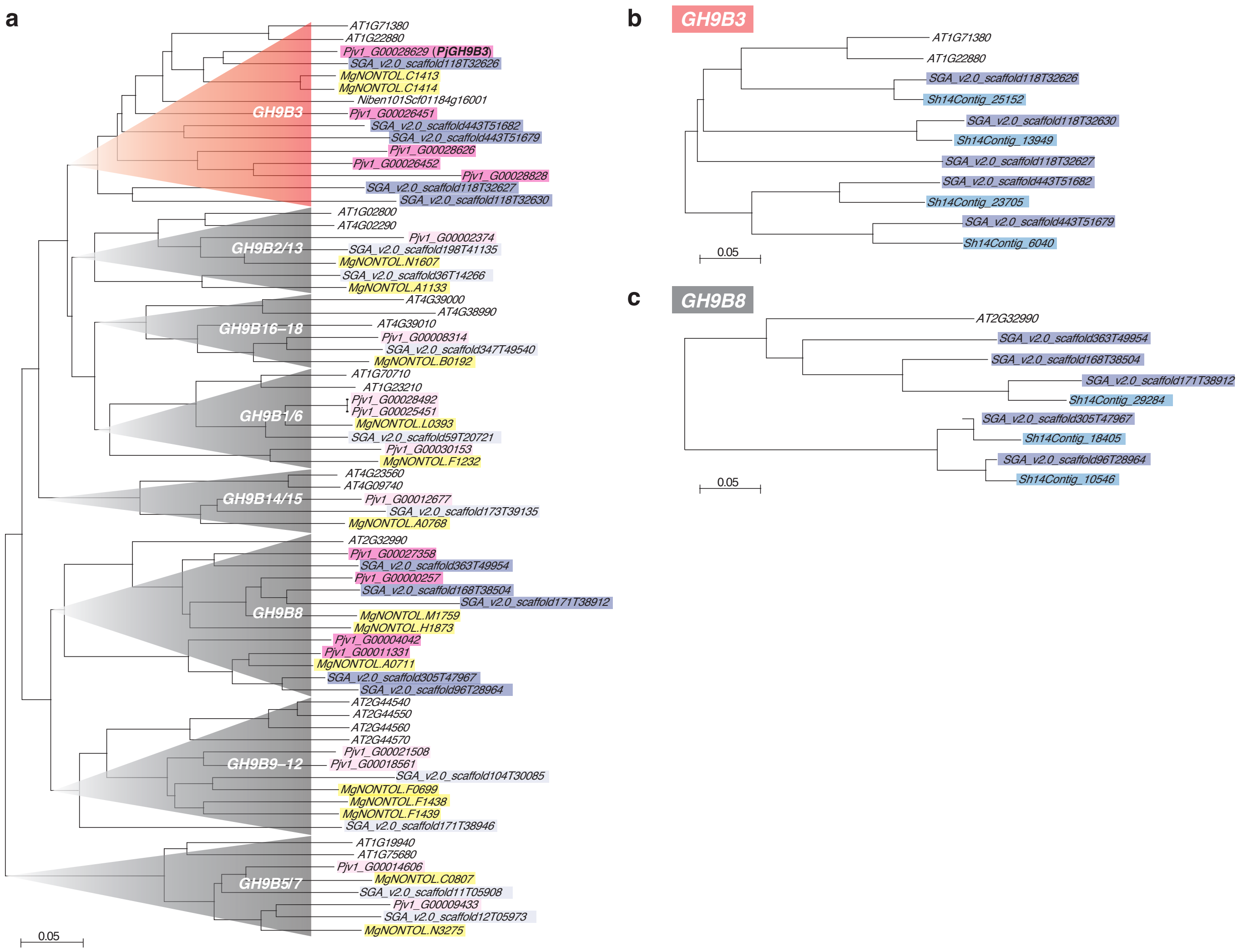

Supplementary Fig. 2: Phylogenetic trees of the *GH9B* gene family for *Arabidopsis*, *P. japonicum*, *S. asiatica*, *E. guttata*, and *S. hermonthica.* **a** Phylogeny of *Glycosyl hydrolase 9B* genes of *E. guttata* (yellow), *P. japonicum* (red), *S. asiatica* (indigo), and *Arabidopsis* reconstructed using deduced amino acid sequences. Genes with less than three genes contained in each clade were given light backgrounds. **b**, **c** Phylogeny of *GH9B3* (b) and *GH9B8* (c) clades of *S. hermonthica* (blue), *S. asiatica* (indigo), and *Arabidopsis.*

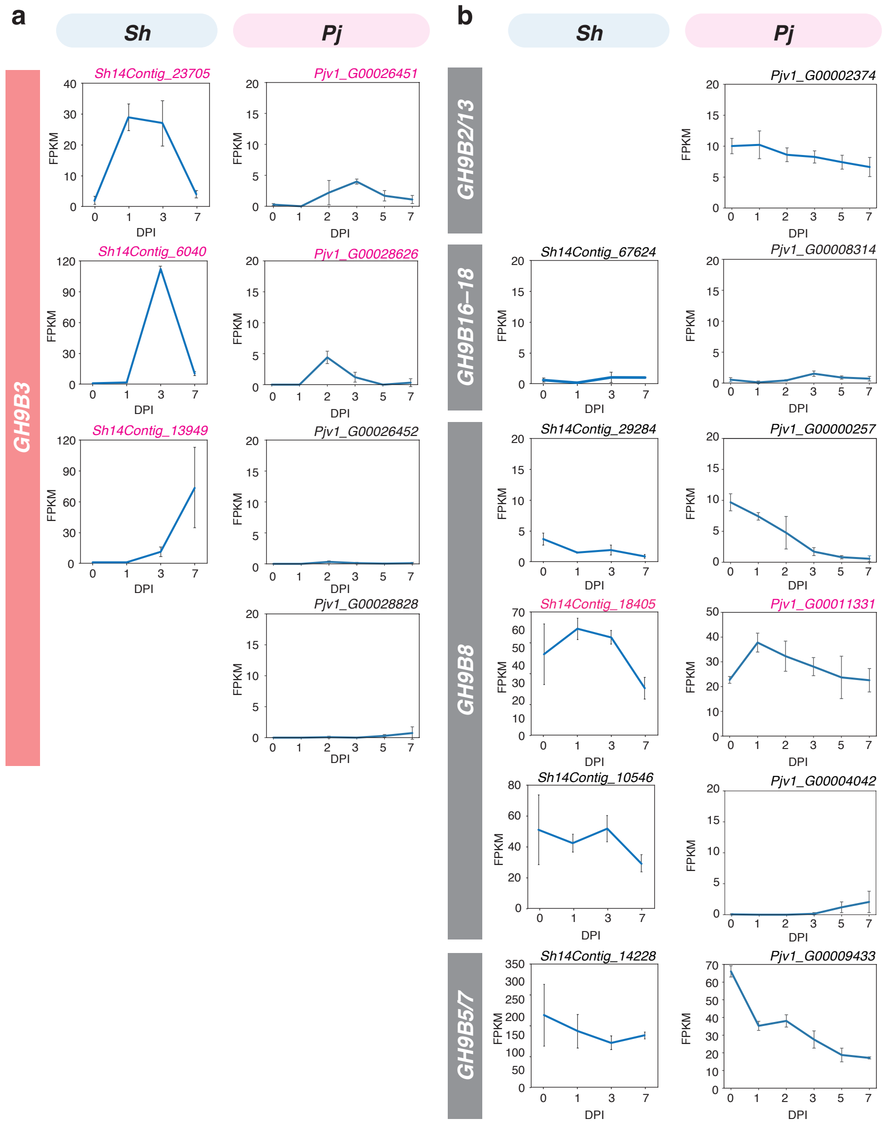

Supplementary Fig. 3: Expression levels of other genes for *Glycosyl hydrolase 9B* family in Orobanchaceae. **a** Expression patterns of other genes of *S. hermonthica* belonging to the GH9B3 clade. **b** Expression patterns of other GH9B genes belonging to clades other than GH9B3 in *S. hermonthica* and *P. japonicum*. *Sh*, *S. hermonthica*; *Pj*, *P. japonicum*.

```
Pjv1_G00026452    1 -----MAFL-NGSQYFLSILLFCLLTFKNSGGDAHDYKDALAKSILFFEGQRSGKLPSMQRMDWRGDSALSDG--AKAGV
Pjv1_G00028828    1 ----------------------------------------------------------------------------MLQV
Pjv1_G00028626    1 -----MAFLKNGSQYYLFILLFCLP-FIIRGGDEYNYKEALTKSILFFEGQRSGELPPNQRLNWRADSALYDG--NEAGV
AT1G22880         1 ----------MASPFFFVFLLSALS-LENTY-ASPNYREALSKSLLFFQGQRSGRLPSDQQLSWRSSSGLSDG--SSAHV
AT1G71380         1 ----------MTSLFFFVLLFSSLL-ISNGD-ANPNYKEALSKSLLFFQGQRSGPLPRGQQISWRASSGLSDG--SAAHV
Pjv1_G00028629    1 MAFLKLSPHFLHLTFFLLFSLTFLSNFTNVH-ASQNYRDALAKSILFFQGQRSGRLPP-QDITWRANSGLSDG--HPAGV
Pjv1_G00026451    1 MAFLNG-----STSFLILFSFTFLCNLRNVQSADHNYKEALTKSIIFLQGQRSGKLPSTQQITWRGDSALSDGEKAGAGV

Pjv1_G00026452   73 DLTGGYYDAGDNVKFNFPMAFTTTMLSWSVIEFGGKLVKTGQLKSAREAVKWGTDYLLKCASKVRSDNKLFVGVANPTLD
Pjv1_G00028828    5 DLTGGYYNVGDNVKFNFPMAFTMTVLSWSVIEYVGQMAKTGQPKSAQEAIRWGTGYLLKCASQVKSRKKLFVGVGDPNRD
Pjv1_G00028626   73 DLTGGYYDSGDNVKFNFPMAFTMTTLSWSVIEFG-SLMERGDLDSAREAIKWGTDYLLKCASQVESHNKLYSGVGDPNKD
AT1G22880        67 DLTGGYYDAGDNVKFNFPMAFTTTMLSWSSLEYGKKMGP--ELQNSRVAIRWATDYLLKCARATPG--KLYVGVGDPNGD
AT1G71380        67 DLTGGYYDAGDNVKFNLPMAFTTTMLSWSALEYGKRMGP--ELENARVNIRWATDYLLKCARATPG--KLYVGVGDPNVD
Pjv1_G00028629   77 DLTGGYYDAGDNVKFNFPMAYTTTMLSWGAIEYGKRLGP--QLQSTRAAIRWGTDYLLKCARATPG--KLYVGVGDPNRD
Pjv1_G00026451   76 NLTGGYYDAGDNVKFNFPMAYTTTMLSWGVIEYGKRLGA--ELQHAREAIRWGTDYLLKCALETPG--KLYVGVANPNLD

Pjv1_G00026452  153 HNCWERPEDMDTDRSVYSVTA---GSDVAGETAAALAAASIVFRKANSSYSKLLRNTANDVMQFAINNKGAYSDQLGGNV
Pjv1_G00028828   85 HQCWERLEDMDTDRSVFSVTA---GSDMAGETAAALAAASIDFKRAELTYSKLLRKTADVVMRFAINNKWAYSDQLRNNV
Pjv1_G00028626  152 HSCWERPEDMDTPRTVYRVTA---GSDLAGEIAAALAAASIVFRKANHRYSEQLRNTAEVVMQFATNNQVSY-----GSV
AT1G22880       143 HKCWERPEDMDTPRTVYSVSPSNPGSDVAAETAAALAASSMVFRKVDPKYSRLLLATAKKVMQFAIQYRGAYSNSLSSSV
AT1G71380       143 HKCWERPEDMDTPRTVYSVSASNPGSDVAAETAAALAAASMVFRKVDSKYSRLLLATAKDVMQFAIQYQGAYSDSLSSSV
Pjv1_G00028629  153 HACWERPEDMDTDRSVYSVSASNPGSDVAAETAAALAAASIVFRRVDPNYSKLLLNTAKNVMQFAISYRGAYSDSLGPYV
Pjv1_G00026451  152 HQCWERPEDMDTDRSVFSVSPSNPGSDVAAETAAALAAASIVFRKVDPRYSKLLRNTAKDVMQFAIDHRGAYSDSLAPYV

Pjv1_G00026452  230 CPFYCSYSGFKDELLWGAAWLYKATGSPVYRNYITSYGENDDVDIFSWDNKFAGANVLLAKEYLVGNNRTFQKFNEKAET
Pjv1_G00028828  162 CPFYCSCLEFKDELLWGEVGLYKATRLPFYRNYITSYGDNDDADILSLDSKFAGANVLLAREYLVGKDRTFEKFSGRAET
Pjv1_G00028626  224 DGFYQSYSGFEDELLWGAAWLYKATNKTSYRNYITSYGCTDDVDTFGWDSKFPGAYVVLAREYLVGKDENFEKFKEKAET
AT1G22880       223 CPFYCSYSGYKDELLWGAAWLHRATNDPYYTNFIKSLGGGDQPDIFSWDNKYAGAYVLLSRRAVLNKDNNFELYKQAAEN
AT1G71380       223 CPFYCSYSGYKDELMWGASWLLRATNNPYYANFIKSLGGGDQPDIFSWDNKYAGAYVLLSRRALLNKDSNFEQYKQAAEN
Pjv1_G00028629  233 CPYYCSYSGYKDELLWGAAWLFRATNLVYYLNFIGSLGANDGTDIFSWDNKYAGARVLLSRRNLVDNDPTFQSFRQQAED
Pjv1_G00026451  232 CPFYCSYSGYKDELFWGAAWLFRATNSVYYYDFIRTLGANDDANIFSWDNKFAGARVLLSR-----------QFSQQAEN

Pjv1_G00026452  310 FMCKILRG--SPSLSIQYTQGGLIH--RLHDSTLQYTTSMTFLITVYAKYLANRNAPKHSFNCNGRGFNSTNLRVQAKAQ
Pjv1_G00028828  242 FMCKILRN--SPSLSIQYTPGDLIH--RLHDNTLQYTTSMTFLITTYAKYIASRNAPKHNFGCRVRVFN-----------
Pjv1_G00028626  304 FMCKILRD--SPSLSIQFTGGGMMYNKNLPDSPLQYVTSLSFLITTYAKYLANRHSPTHKIDCEGHAFNPKKLRNQAKAQ
AT1G22880       303 FMCKILPN--SPSSSTKYTKGGLMY--KLPQSNLQYVTSITFLLTTYAKYMK---STKQTFNCGNSLIVPNALINLSKRQ
AT1G71380       303 FICKILPD--SPSSSTQYTQGGLMY--KLPQSNLQYVTSITFLLTTYAKYMK---ATKHTFNCGSSVIVPNALISLSKRQ
Pjv1_G00028629  313 FMCRILPNSYSRSSSTQYTPGGLMY--KMDASNLQYVTSITFLMTTYSKYMA---TTKHTFNCGNLPITSNILRTLAKKQ
Pjv1_G00026451  301 FMCKVLPNS-PYLSDTQYTKGGLMY--KLDGSNLQYVTAITFLFTTYAKYLANRNSPKHSFTCGGQIYTASYLRVVAKSQ

Pjv1_G00026452  386 VDYILGTNRMKMSYMVGYGPKYPMRVHHRGASLP----LGKKISCGDGF-TYLHTPNPNPNILTGAIVGGPDENDFYKDN
Pjv1_G00028828  307 -------------------PTYS---------------------------------------------------------
Pjv1_G00028626  382 VDYILGTNPMKTSYMVDYGPIFPERVHHRAASSPSVRAHPQHIACGVGFGLYLHTYNPNPNTLTGAIVGGPDWNDVYLDD
AT1G22880       376 VDYVLGVNPMKMSYMVGFSSNFPKRIHHRGSSLPSRAVRSNSLGCNGGF-QSFRTQNPNPNILTGAIVGGPNQNDEYPDQ
AT1G71380       376 VDYILGDNPIKMSYMVGFSSNFPKRIHHRASSLPSHALRSQSLGCNGGF-QSFYTQNPNPNILTGAIVGGPNQNDGYPDQ
Pjv1_G00028629  388 VDYILGDNPMKMSYMVGYGQSFPKRIHHRGSSLPSRNVSPQSFGCDGGK-QFFYSPNPNPNTLTGAIVGGPNHNDFFADE
Pjv1_G00026451  378 VDYILGDNPMKMSYMVDYTKNFPKRIHHRGSSVPSIIDHPKKITCGEGF-SYLQTSSANPNILTGAIVGGPDRNDRYADD

Pjv1_G00026452  461 RDEYSQSEPATYINAPLVGTLAYFAAKQK-
Pjv1_G00028828      ------------------------------
Pjv1_G00028626  462 RSSYSQSEPATYINAPLVGILAYFAGKKQ-
AT1G22880       455 RDDYTRSEPATYINAAFVGPLAYFAASRSP
AT1G71380       455 RDDYSHAEPATYINAAFVGPLAYFAAGRST
Pjv1_G00028629  467 RTDYSHSEPATYINAAIVGPLAYFAGVN--
Pjv1_G00026451  457 RNNFGQSEPTTYINAAIVGPLAYLAAKQ--
```

Supplementary Fig. 4: Alignment analysis of amino acid sequences for genes of the GH9B3 clade in *Arabidopsis* and *P. japonicum*. Amino acid sequence alignments were performed on genes of *Arabidopsis* and *P. japonicum* GH9B3 clade using clustal2.1 software. The amino acid residues highlighted in tangerine and magenta indicate a catalytic domain and *O*-glycosylation sites, respectively. Red line indicates the secretory signal peptides.

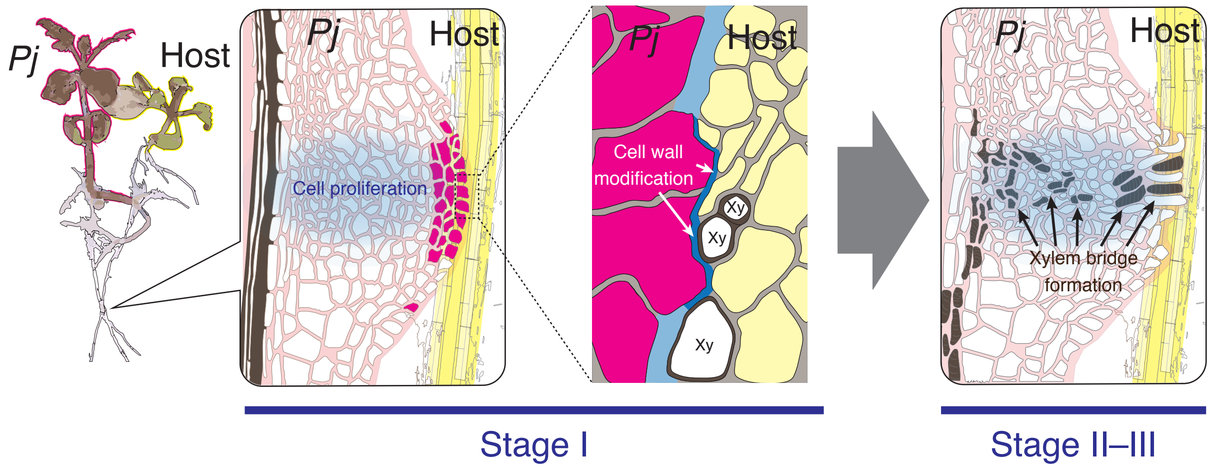

Supplementary Fig. 5: Model of parasitism by *P. japonicum*. Upon detection of host plant roots, *P. japonicum* generates haustoria where cells are proliferated through cell division (colored in blue shade). *PjGH9B3* is induced at the periphery of the haustorium (colored in magenta) to facilitate tissue adhesion when in contact with the host plant root (stage I). In magnified illustration, the interface between cells of *P. japonicum* and host plant is marked in blue and especially thin cell wall region through cell wall modification is marked in dark blue. Subsequently, a xylem bridge (colored in brown) is formed between *P. japonicum* and the host plant root (stages II–III). Arrows indicate decreased cell wall thickness at the interface between parasitic and host tissues. *Pj*, *P. japonicum.* Xy, Xylem.
