# Supplementary material for: Host-parasite tissue adhesion by a secreted type of β-1,4-glucanase in the parasitic plant Phtheirospermum japonicum
Source: Commun Biol. 2020 Jul 30;3:407. doi: 10.1038/s42003-020-01143-5 (PMC7393376; doi:10.1038/s42003-020-01143-5)
Supplement: Supplementary file 2 — Description of Additional Supplementary Files [file 42003_2020_1143_MOESM2_ESM.pdf]

Description of Additional Supplementary Files

File Name: Supplementary Data 1
Description: Grafting experiments performed with a root-parasitic plant, P. japonicum.

File Name: Supplementary Data 2
Description: Expression data for parasitism between P. japonicum and Arabidopsis.

File Name: Supplementary Data 3
Description: Expression data for grafting between P. japonicum and Arabidopsis.

File Name: Supplementary Data 4
Description: List of GO terms in six clusters shown in Supplementary Fig. 1a (BP category, $P < 0.01$).

File Name: Supplementary Data 5
Description: List of GO terms in six clusters shown in Supplementary Fig. 1a (CC category).

File Name: Supplementary Data 6
Description: List of GO terms in six clusters shown in Supplementary Fig. 1a (MF category, $P < 0.01$)

File Name: Supplementary Data 7
Description: List of genes overlapping between parasitism and grafting of P. japonicum shown in Fig. 4c.

File Name: Supplementary Data 8
Description: List of GO terms in genes overlapping between parasitism and grafting of P. japonicum shown in Fig. 4c (BP category).

File Name: Supplementary Data 9
Description: List of GO terms in genes overlapping between parasitism and grafting of P. japonicum shown in Fig. 4c (CC category).

File Name: Supplementary Data 10
Description: List of GO terms in genes overlapping between parasitism and grafting of P. japonicum shown in Fig. 4c (MF category).

File Name: Supplementary Data 11
Description: Primers used in this study

File Name: Supplementary Data 12
Description: Data for Fig. 7f.

File Name: Supplementary Data 13

Description: Data for Fig. 7i.
